# Supplementary material for: Melanin-Based Nanoparticles for Lymph Node Tattooing: Experimental, Histopathological and Ultrastructural Study
Source: Nanomaterials (Basel). 2024 Jul 4;14(13):1149. doi: 10.3390/nano14131149 (PMC11243654; doi:10.3390/nano14131149)
Supplement: Supplementary file 1 [file nanomaterials-14-01149-s001.zip › nanomaterials-3068064-supplementary.pdf]

Supplementary information

Table S1. Ease of identification of the tattooed lymph nodes. According to the identification scale described in Figure 2 of the main body of the manuscript.

Lymph node	Study	Concentration	Ease of identification
1	Short-term	20 mg/ml	3
2	Short-term	20 mg/ml	3
3	Short-term	20 mg/ml	3
4	Short-term	20 mg/ml	1
5	Short-term	20 mg/ml	3
6	Short-term	20 mg/ml	3
7	Short-term	20 mg/ml	3
8	Short-term	20 mg/ml	1
9	Short-term	20 mg/ml	2
10	Short-term	20 mg/ml	1
11	Long-term	20 mg/ml	3
12	Long-term	20 mg/ml	3
13	Long-term	20 mg/ml	3
14	Long-term	20 mg/ml	2
15	Long-term	20 mg/ml	3
16	Long-term	20 mg/ml	1
17	Long-term	10 mg/ml	3
18	Long-term	10 mg/ml	2
19	Long-term	10 mg/ml	1
20	Long-term	10 mg/ml	3
21	Long-term	10 mg/ml	1
22	Long-term	10 mg/ml	2
23	Long-term	10 mg/ml	2
24	Long-term	10 mg/ml	1
25	Long-term	10 mg/ml	1

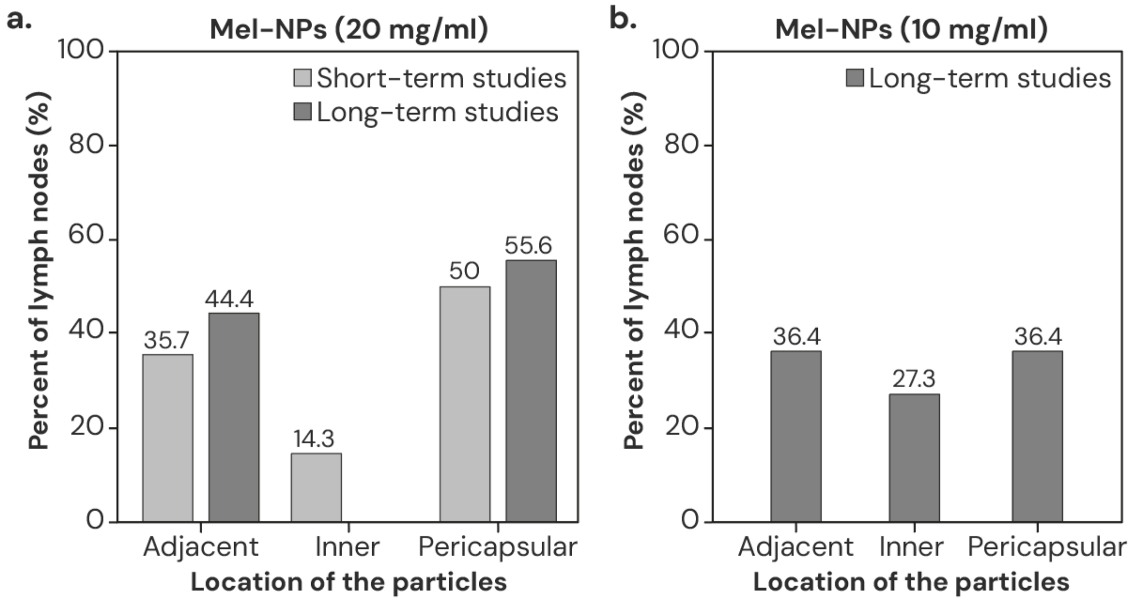

Figure S1. Percentage of lymph nodes with adjacent, inner or pericapsular localization of Mel-NPs.

Table S2. Localization of Mel-NPs in the lymph nodes. According to the localization description showed in Figure 2 of the main body of the manuscript.

			**Location**		
**Lymph node**	**Study**	**Concentration**	**Adjacent**	**Pericapsular**	**Inner**
1	Short-term	20 mg/ml	x	x	
2	Short-term	20 mg/ml	x	x	
3	Short-term	20 mg/ml			x
4	Short-term	20 mg/ml	x		
5	Short-term	20 mg/ml	x	x	
6	Short-term	20 mg/ml	x	x	x
7	Short-term	20 mg/ml		x	
8	Short-term	20 mg/ml		x	
9	Short-term	20 mg/ml		x	
10	Short-term	20 mg/ml	*Mel-NPs were not identified*		
11	Long-term	20 mg/ml	x	x	
12	Long-term	20 mg/ml	x	x	
13	Long-term	20 mg/ml	x	x	
14	Long-term	20 mg/ml	x	x	
15	Long-term	20 mg/ml		x	
16	Long-term	20 mg/ml	*Mel-NPs were not identified*		
17	Long-term	10 mg/ml	x	x	
18	Long-term	10 mg/ml	x	x	x
19	Long-term	10 mg/ml			x
20	Long-term	10 mg/ml	x	x	
21	Long-term	10 mg/ml			x
22	Long-term	10 mg/ml	x		
23	Long-term	10 mg/ml		x	
24	Long-term	10 mg/ml	*Mel-NPs were not identified*		
25	Long-term	10 mg/ml	*Mel-NPs were not identified*		

Table S3. Extent of inflammation maximum length in the lymph nodes. Note that some nodes were not counted due to rupture of nodal tissue during surgical resection or sample processing.

**Lymph node**	**Study**	**Concentration**	**Extent of inflammation**
1	Short-term	20 mg/ml	265 μm
2	Short-term	20 mg/ml	221 μm
3	Short-term	20 mg/ml	487 μm
4	Short-term	20 mg/ml	205 μm
5	Short-term	20 mg/ml	1076 μm
6	Short-term	20 mg/ml	592 μm
7	Short-term	20 mg/ml	153 μm
8	Short-term	20 mg/ml	316 μm
9	Short-term	20 mg/ml	264 μm
10	Short-term	20 mg/ml	-
11	Long-term	20 mg/ml	548 μm
12	Long-term	20 mg/ml	611 μm
13	Long-term	20 mg/ml	828 μm
14	Long-term	20 mg/ml	258 μm
15	Long-term	20 mg/ml	355 μm

16	Long-term	20 mg/ml	-
17	Long-term	10 mg/ml	490 µm
18	Long-term	10 mg/ml	84 µm
19	Long-term	10 mg/ml	213 µm
20	Long-term	10 mg/ml	224 µm
21	Long-term	10 mg/ml	388 µm
22	Long-term	10 mg/ml	358 µm
23	Long-term	10 mg/ml	189 µm
24	Long-term	10 mg/ml	-
25	Long-term	10 mg/ml	-

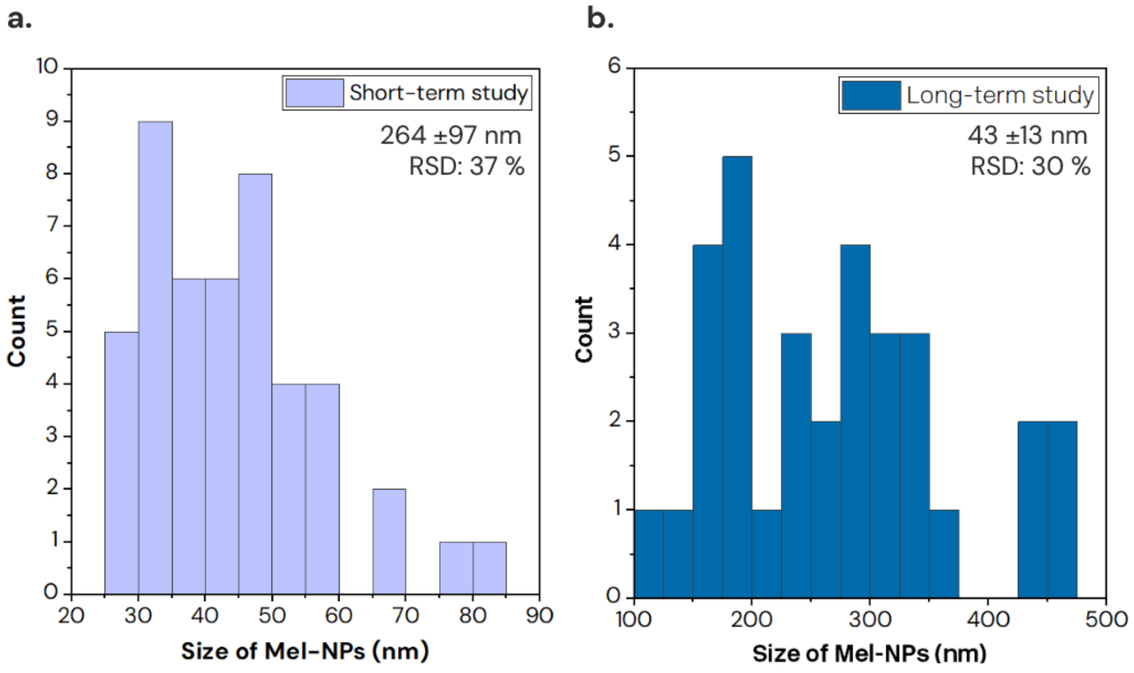

Figure S2. Diameter distribution histograms of Mel-NPs in the short and in long-term studies *in vivo*.
